# Virtual reality set-up for studying vestibular function during head impulse test

**DOI:** 10.3389/fneur.2023.1151515

**Published:** 2023-03-29

**Authors:** Clément Desoche, Grégoire Verdelet, Romeo Salemme, Alessandro Farnè, Denis Pélisson, Caroline Froment, Ruben Hermann

**Affiliations:** ^1^Université Claude Bernard Lyon 1, CNRS, INSERM, Centre de Recherche en Neurosciences de Lyon CRNL U1028 UMR5292, Neuro-Immersion Platform, Bron, France; ^2^Université Claude Bernard Lyon 1, CNRS, INSERM, Centre de Recherche en Neurosciences de Lyon CRNL U1028 UMR5292, IMPACT, Bron, France; ^3^Hospices Civils de Lyon, Neuro-Ophthalmology Unit, Hopital Neurologique et Neurochirurgical P Wertheimer, Bron, France; ^4^Hospices Civils de Lyon, ENT, Cervico-Facial Surgery and Audiophonology, Hôpital Edouard Herriot, Lyon, France

**Keywords:** vestibular function, head impulse test, virtual reality, vestibulo-ocluar reflex, visio-vestibular mismatch, visual feedback

## Abstract

**Objectives:**

Virtual reality (VR) offers an ecological setting and the possibility of altered visual feedback during head movements useful for vestibular research and treatment of vestibular disorders. There is however no data quantifying vestibulo-ocular reflex (VOR) during head impulse test (HIT) in VR. The main objective of this study is to assess the feasibility and performance of eye and head movement measurements of healthy subjects in a VR environment during high velocity horizontal head rotation (VR-HIT) under a normal visual feedback condition. The secondary objective is to establish the feasibility of VR-HIT recordings in the same group of normal subjects but under altered visual feedback conditions.

**Design:**

Twelve healthy subjects underwent video HIT using both a standard setup (vHIT) and VR-HIT. In VR, eye and head positions were recorded by using, respectively, an imbedded eye tracker and an infrared motion tracker. Subjects were tested under four conditions, one reproducing normal visual feedback and three simulating an altered gain or direction of visual feedback. During these three altered conditions the movement of the visual scene relative to the head movement was decreased in amplitude by 50% (half), was nullified (freeze) or was inverted in direction (inverse).

**Results:**

Eye and head motion recording during normal visual feedback as well as during all 3 altered conditions was successful. There was no significant difference in VOR gain in VR-HIT between normal, half, freeze and inverse conditions. In the normal condition, VOR gain was significantly but slightly (by 3%) different for VR-HIT and vHIT. Duration and amplitude of head impulses were significantly greater in VR-HIT than in vHIT. In all three altered VR-HIT conditions, covert saccades were present in approximatively one out of four trials.

**Conclusion:**

Our VR setup allowed high quality recording of eye and head data during head impulse test under normal and altered visual feedback conditions. This setup could be used to investigate compensation mechanisms in vestibular hypofunction, to elicit adaptation of VOR in ecological settings or to allow objective evaluation of VR-based vestibular rehabilitation.

## Introduction

The vestibulo-ocular reflex (VOR) is responsible for stabilizing vision during head movements. Due to its multiple sensorineural organs and broad operating frequencies, the vestibular system is usually tested by a multitude of tools. In the past decades, high-speed recording of eye and head movements during head impulses has become an increasingly popular way to determine vestibular function and/or deficit. The so-called video Head Impulse Test (vHIT) (1) allows for an objective measurement of the vestibulo-ocular reflex (VOR) during head rotation at physiological frequencies. This test has also given insight in oculomotor compensation following vestibular deficit by demonstrating different types of catch up saccades (2). Due to its reliability and ease of use, the vHIT has become a reference tool in clinical use but also in the scientific literature when studying horizontal canal function during head impulses.

The plasticity of the VOR with regards to changes of visual feedback has been known and studied for several decades by using tools such as prisms (3), magnifying spectacles or optokinetic drums (4, 5) and lasers (6). These experiments have highlighted the ability of the VOR gain not only to be adaptively recalibrated but also to adapt in a context specific manner (7). Nevertheless, these tools have limitations, which depend on physical parameters (i.e., power of refraction in prisms, frequency of head movements) or on unecological setups (motorized turn-table, optokinetic drum).

Virtual reality (VR) offers a nowadays-convenient means to overcome these limitations. The commercialization of several different types of VR goggles in recent years has allowed a greater democratization of this tool. Virtual reality also allows for new possibilities of rehabilitation in patients with vestibular impairment (8, 9). VR has also been employed in some scientific studies focusing on balance or spatial navigation in patients with bilateral vestibular loss (10, 11). Its ability to create rich, life-like, immersive environments and to manipulate visual information makes it a very promising tool in vestibular research.

As of yet there is however no study focusing on head and eye movements recording in a virtual reality setup for the quantification of the vestibular function. Combining vHIT and VR could provide a new tool for studying vestibular adaptation in healthy subjects, but also for bringing new insight in vestibular compensation in people with vestibular deficit.

The main objective of the present study is to assess the feasibility and performance of eye and head movement measurements of healthy subjects in a VR environment during high velocity horizontal head rotation (VR-HIT) under a normal visual feedback condition. Such VR-HIT measurements will be compared to classical vHIT measurements in the same participants. The secondary objective is to establish the feasibility of VR-HIT recordings in the same group of normal subjects but under altered visual feedback conditions.

## Materials and equipment

This prospective study was held in the Neuro-Immersion platform of the Lyon Neuroscience Research Centre between December 2020 and October 2021.

### Subjects

Inclusion criteria were healthy participants with an age between 18 and 90. We chose this wide range so that our cohort would be representative of patient population. Exclusion criteria were: underlying neurologic, vestibular or otologic disorder, disabling motion sickness, best corrected visual acuity lower than 5/10, ocular motor palsy, ocular instability in primary gaze position, instability of the cervical spine and if they had taken drugs interfering with eye movements. Absence of exclusion criteria were verified by thorough anamnesis prior to the inclusion visit.

A total of 12 healthy participants (5 males, 7 females) were included. Mean age was 52.8 (SD 14, min 30, max 74).

### Ethical issue

All participants were informed about the design and purpose of the study, and all gave their informed, written consent to the protocol. Approval was received from the National French ethical committee on human experimentation (ID-RCB: 2020-A00184-35), in agreement with French law (March 4, 2002) and the Declaration of Helsinki. The study was registered in a public trials registry (ClinicalTrials.gov Identifier: NCT04268615).

### Video head impulse test device

The vHIT was recorded using a lightweight portable vHIT device (Hardware: ICS Impulse, GN Otometrics, Taastrup, Denmark, Software: Otosuite Vestibular software) (1). Head movements were recorded with a nine-axis motion sensor, and movements of the right eye were recorded with a high-velocity infrared camera. Both the motion-sensor and camera were mounted on a lightweight eye frame and had a sampling rate of 250 Hz. Eye movements were calibrated by having the subject gaze toward light spots projected at eye level on the wall from two lasers build in the eye frame.

### Virtual reality and eye-head motion tracking devices

• Virtual reality Head Mounted Device

For virtual reality (VR), we used the HTC Vive® (HTC Europe Co. Ltd., Salamanca, Wellington Street, Slough, Berkshire SL1 1YP, U.K. Company registration number: 04826012). This VR head mounted device (HMD) was equipped with Dual AMOLED lenses with a resolution of 1,080 × 1,200 pixels per eye and a refresh rate of 90 Hz, covering a field of view of 110°. The HMD position and rotation were measured by two complementary integrated tracking systems: a form of dead reckoning tracking with an internal Inertial Measurement Unit composed by a gyroscope and an accelerometer which allows for high update rates, and a lighthouses BASE stations which emits infrared light allowing for a high accuracy (12).

• Vicon Head motion tracking

**Figure 1:**
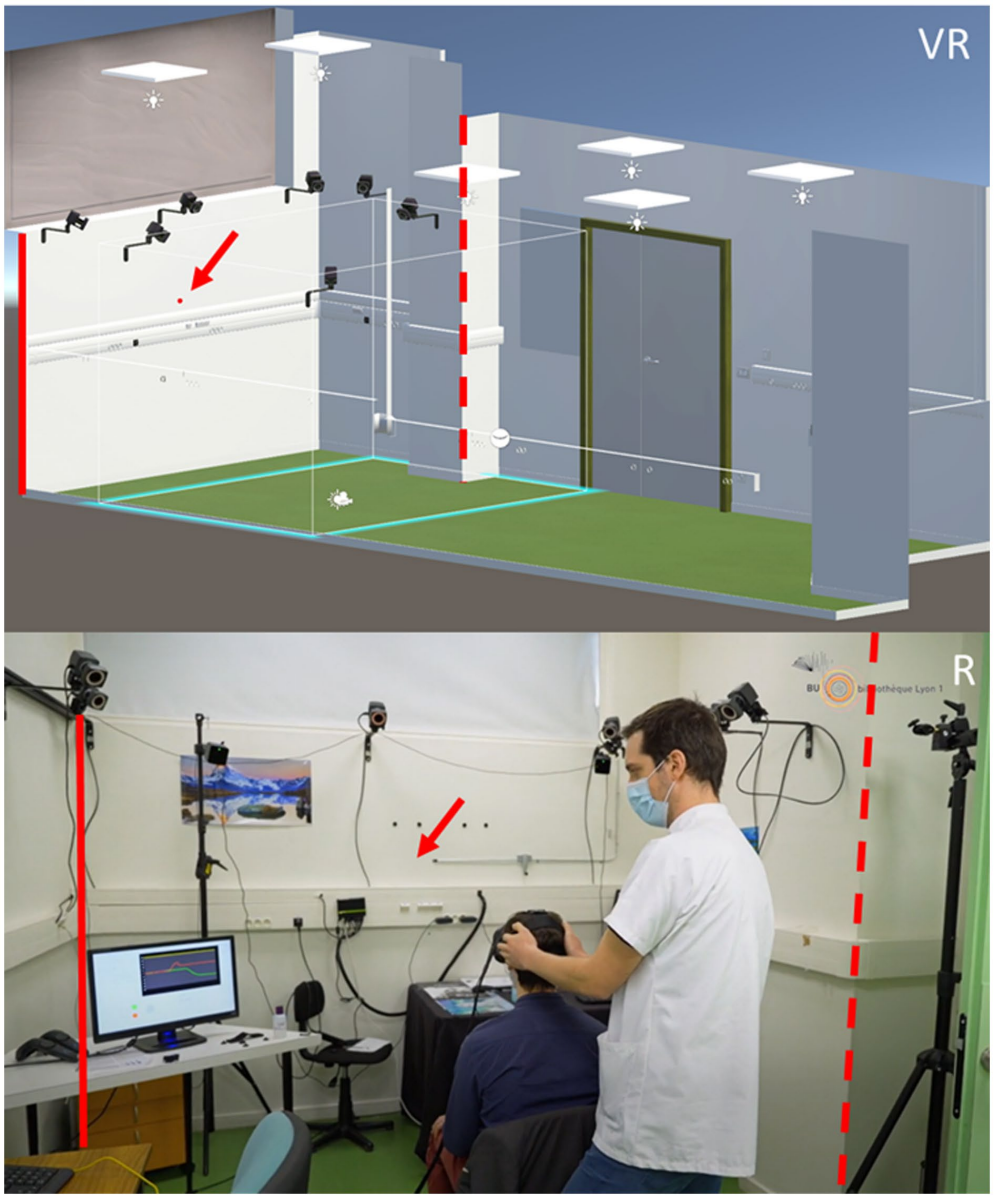
Simulated environment in virtual reality (VR) and picture of the real room (R) where the virtual reality experiments were performed. The size of the real room as well as some of its features (doors, cameras, power plugs…) were faithfully represented in the VR environment. The full and dotted red line are placed on the same corners in the virtual and real room to help comparisons. The red arrow points to where the fixation point is (VR) or should be (R).

As the two integrated head tracking systems described above produced artifacted signals due to the high head velocities (>200 °/s) (see section “Results”), we additionally used a VICON® motion capture system (Oxford Metrics plc, Oxford, United Kingdom) with a submillimetric accuracy [between 0.06 and 0.3 mm in static or dynamic conditions (13, 14)]. We used a setup of 7 Bonita cameras (resolution of 1 megapixel), with the Vicon Tracker® software 2.0.1, to track reflective markers illuminated by near infrared light. The cameras were positioned in an arc around the subject viewing the head from above (Figure 1). This set-up allowed us to acquire the head motion with a frequency of 250 Hz and assure the compatibility with the Vive Lighthouses IR system as confirmed in a previous study (15).

Reflective markers were fixed to both the HMD and a cap on the subject’s head, allowing us to measure simultaneously and compare the head motion and the HMD motion.

• SMI Eye Tracking

For eye tracking, we used the SMI® Eye tracking device (SensoMotoric Instruments Gesellschaft für innovative Sensorik mbH Warthestr. 21, 14,513 Teltow, Germany), which was implemented in the HTC Vive HMD. This system provided average eye position data of both eyes at a frequency of 250 Hz with a spatial accuracy of 0.67 ° and a spatial precision of 0.1° (12). The eye position signal was calibrated by using the 5-points calibration procedure of the constructor’s software. We used the SMIEyeTrackingUnity Software Development Kit - with the default initialization variables - and the native asynchronous callback function to collect every new eye data samples as soon as generated by the eye tracker.

• Programming

The virtual environment was build *via* the Game Engine Unity® version 2019.4.26f1 (Unity Technologies, San Francisco, US), operating with the Steam VR Software Development Kit to control the VR HMD HTC Vive. The Vicon Tracker 2.0.1 was linked to Unity with the DataStream® program (Oxford Metrics plc, Oxford, United Kingdom).

A VR-ready computer with Windows 10 ×64 equipped with an Intel® Core® i7-7700K CPU @ 4,20GHz, 16GB of RAM (Intel, Santa Clara, United-States of America), and a NVIDIA® GeForce GTX 1060 with 6GB (NVIDIA, Santa Clara, United-States of America), was used for the experiments. Another computer managing VICON data acquisition was equipped with an Intel® Core™ i7-4,771 CPU @ 3.5 GHz, 8 GB of RAM, and a NVIDIA® Quadro K2000 (NVIDIA, Santa Clara, United-States of America). Head and eye tracking data were sent in real time to the game engine. The data was then synchronized by using the computer’s internal clock as a common time reference. This clock was also used to set the exact moment when the signal was given to the experimenter to start the trial and when data recording began.

## Method

### Video head impulse test

The method for this evaluation has been described in detail in a previous study (16). The vHIT was performed before the VR-HIT. Outward horizontal head impulses were performed by a single experienced examiner standing behind the patient. The fixation point was located 2 meters in front of the subject. The head was tilted forward to align the plane of the horizontal semicircular canals with the horizontal plane. A minimum of 10 valid horizontal head impulses with a target speed >200°/s were realized in each direction. Head and eye velocity data were then exported in CSV format for offline analysis.

### Virtual reality (VR-HIT)

• Simulated environment

Participants were seated and immersed in a virtual version of the room in which the experiment was actually conducted (Figure 1). A red dot was added in this virtual room at a distance of 2 m in front of the subject and used as a fixation point.

• Visual feedback conditions

### Normal (A)

In the normal condition, the head rotation leads to a rotation of the visual scene displayed in the HDM, of the same size but in the opposite direction. This condition corresponds to the situation in real life and in vHIT.

### Half (B)

In the half condition, the head rotation leads to a rotation of the virtual visual scene in the opposite direction but of only half of its size, as if the HMDs motion was divided by two.

To achieve this, the visual display in the HMD was driven by the half-attenuated value of the horizontal angular displacement between the current and starting positions of the HMD.

### Freeze (C)

In the freeze condition, the image in the HMD remained fixed regardless of the head movement, cancelling out any visual feedback of head movement. To achieve this, the visual display in the HMD was driven by the exact value of the angular displacement between the current and starting positions of the HMD.

### Inverse (D)

In the inverse condition, the head rotation leads to a rotation of the virtual visual scene of the same size and in the same direction. This condition mimics the visual information that would have been generated during a head rotation in the opposite direction.

To achieve this, the visual display in the HMD was driven by the opposite value of the horizontal angular displacement between the current and starting positions of the HMD.

• Trials

**Figure 2:**
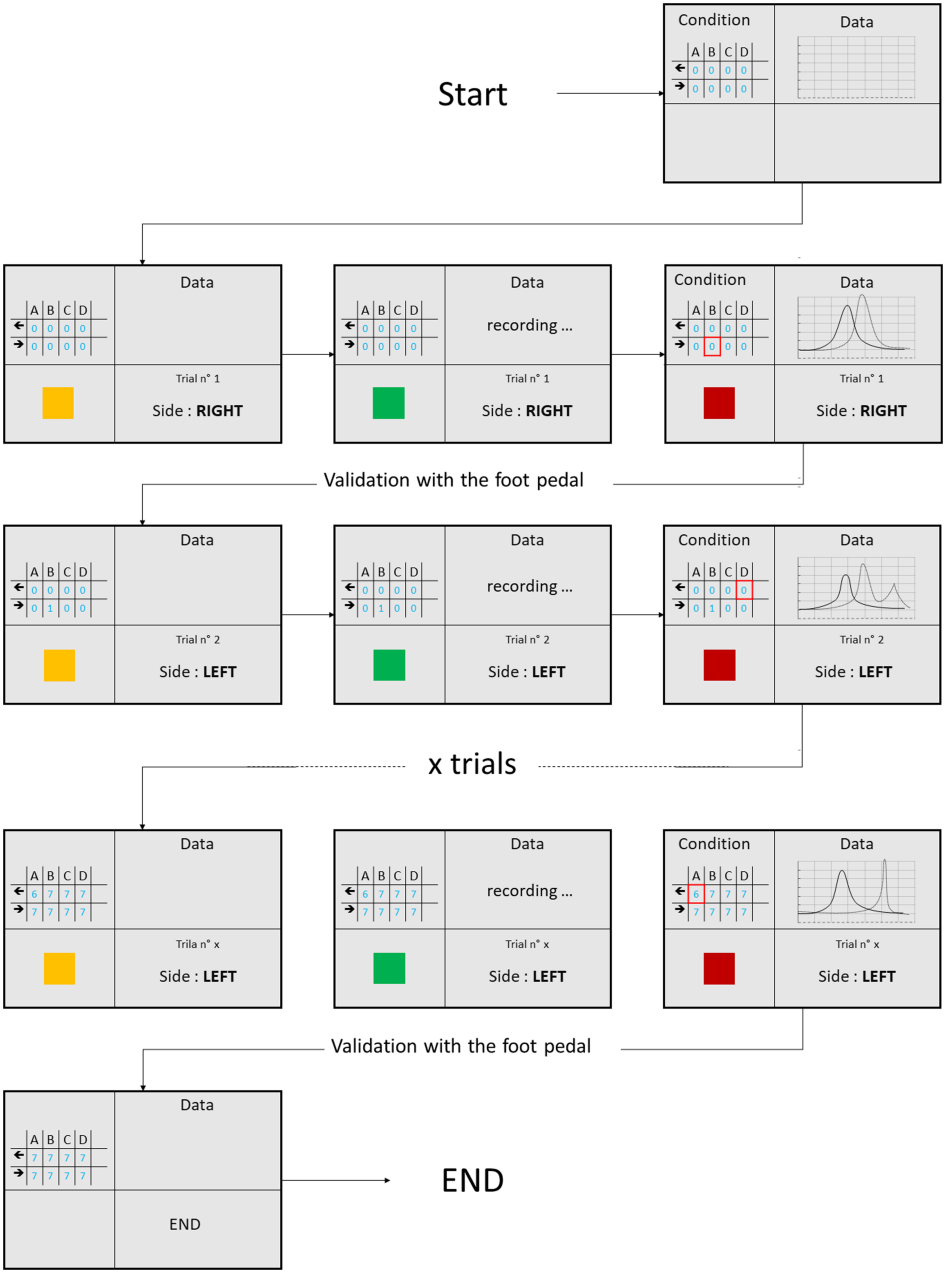
Display of screen N°1 during the protocol. The lower left side of the screen indicates the action the examiner must do. Yellow: prepare for head impulse; Green: Head impulse; Red; no head movement. The lower right side indicates the randomized direction of the head impulse as well as the total number of trials. The upper right side shows recordings of head and eye position during the last trial. The upper left side of the screen shows the progression of validated trials for the four different conditions (A: normal; B: Freeze; C: Inverse; D Half), leftward and rightward arrows indicate the direction of head rotation. The test ended as soon as seven head impulses for each direction and each condition have been validated by the examiner.

The examiner attempted to rotate the head in exactly the same way as for vHIT. Standing behind the participant, the examiner first tilted the participant’s head forward to align the plane of the horizontal semicircular canals with the horizontal plane and positioned the participant’s head horizontally to re-align it with the central fixation point. He then induced centrifugal horizontal head impulses, without knowing under which one of the 4 conditions the current trial was performed (random sequence) until after its completion. Two screens were visible to the examiner. Screen N°1 was divided in 4 sections showing information relative to the current trial (Figure 2). Screen N°2 showed what the subject could see during the head set. Horizontal head impulses were performed in both directions, according to randomized order.

Seven validated head thrusts per direction and per condition had to be performed by the end of the protocol. The 56 different trials were presented with different randomized orders between subjects in order to avoid anticipation by the examiner. To validate a trial, the examiner could look at screen N°1 where recordings of head and eye positions during the head impulse test were shown. If he detected no artefact, the examiner pressed twice on the foot pedal in order to validate the trial, whereas in case of artefact (i.e., eye blink, head rotation performed before the recording started…), he pressed the foot pedal once in order to reject the trial. Rejected trials were then queued again to be replayed at the end of the initially randomized 56 trials. The protocol ended when 56 validated head impulses (7 per condition and per direction) were recorded.

As shown in Figure 2, once the information regarding the direction and the condition appeared on screen N°1, the examiner had between 1,500 and 2,500 ms to prepare (yellow square) before the recording would start (green square). The duration of the data recording period for every trials was 2000 ms. The specific visual feedback for any trial started after the examiner pressed the food pedal to initiate the trial and ended after the examiner validated or rejected the trial. After this the visual feedback returned to normal end the head was rotated back to the center until the beginning of the next trial. This time interval includes the above mentioned 1,500–2,500 ms, the 2000 ms of data recording and the time it took the examiner to decide regarding the trial.

At the end of the protocol, head and eye movements were exported in Comma-Separated Values format (.CSV) for off-line analysis.

### Analysis of head and eye movements

Data analysis for both vHIT and VR-HIT was done in a program developed in our lab running on MATLAB v.8.1 (MathWorks, MA, USA). Details of this analysis can be found in a previous article (16). To sum up, five cursors were used to identify in each movement: the starting and ending positions, the onset and ending times as well as the time of maximum velocity. These cursors were first positioned automatically based on a threshold of head and eye velocity (5°/s) to differentiate movements from artefacts. Each automatically-detected movement was checked and cursors could be manually adjusted. We chose to identify a maximum of four eye movements per head impulse (vestibulo-ocular reflex and up to 3 catch up saccades). Covert saccades (CS) were defined as saccades occurring before the end of the head movement and overt saccades (OS) were those occurring after the end of the head movement (2). For each head impulse, VOR gain was calculated as: (amplitude of slow eye movement)/(amplitude of concurrent head movement). Saccadic gain was calculated as: (amplitude of eye movement during CS or OS)/(amplitude of total head movement). Latencies were calculated as the onset time of each eye movement (VOR, CS, OS) relative to the beginning of head movement. For each subject, the occurrence of CS was determined in percent as the total number of CS relative to the total number of head impulses. The consistency of CS initiation was determined by the mean of individual standard deviation of the latency.

In order to ensure the most rigorous analysis of eye movements to compare vHIT and VR-HIT in the normal condition (A), a recalibration was done in both vHIT and VR-HIT. We assumed that 500 ms after the beginning of the head movement the eye should be aligned accurately with the target. Thus the eye position signal was gain-adjusted such that the total eye movement (VOR and saccade(s) if present) equals the total head movement. We thus refer in the results to both “raw” and “recalibrated” eye data.

### Statistical analyses

All data were stored and analyzed using JASP (JASP Team (2022). JASP (Version 0.16.2)). Statistical analysis was done using paired samples T-Test, Wilcoxon signed-rank test, Chi^2^, Fishers exact test or ANOVA depending on the normality of the distribution and the number of variables tested. All tests were two-tailed and *p*-values <0.05 were considered significant.

## Results

### Head tracking

**Figure 3:**
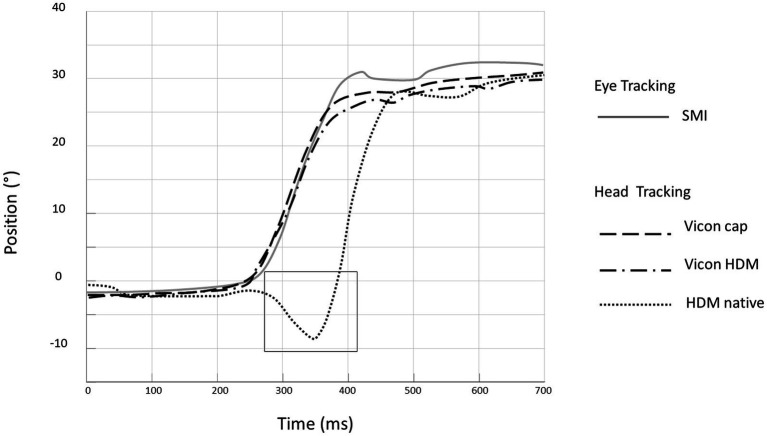
Comparison of three head measurement systems: example of eye and head movements during a single head impulse test in the rightward direction. Eye position is measured by the SMI system (inverted in the plot by convention: grey line). Head position (black lines) is measured simultaneously by the Native Vive system (dotted line ^……^) and the Vicon system using markers fixed on the cap (dashed line ^_ _ _^) and and on the HMD (dash-dotted line ^_ . _ . _^). The black rectangle highlights the artefact occurring with the native Vive system.

During preliminary trials, we compared head movements measurements provided by the VR system and the Vicon system (tracking markers fixed both on a Cap and on the HMD). When looking at the head positional data delivered by the VR system, a large and consistent artefact occurred during the movement, despite pre- and post-movement positions similar to those delivered by the two Vicon-based systems. In contrast, the two head position data delivered by the Vicon system were very similar, revealing no slippage of the HDM, and were consistent with what can be expected from the eye-movement response. Figure 3 displays a comparison of all three head measurement systems.

Thus, for the rest of the study, all head motion data were measured by the VICON system using reflective markers fixed directly on the HMD.

### General data for vHIT and VR-Hit

For vHIT the mean number of head impulses per side and per subject was 15.75 (SD 0.01).

In VR, the mean number of trials per subject and across all 4 conditions was 74 (S.D. 7.7), namely 17, 18, 19 and 20 for the Half, Inverse, Freeze and Normal conditions, respectively. As the minimum number of validated trials was 56 (see Methods), then approximately 25% of trials were rejected during recording of VR-HIT. The mean duration of the VR-HIT protocol was 26 min (S.D. 4 min). Mean trial length was thus 34 s. No breaks were needed for subjects during the VR-HIT protocol. No difference in performance was noted between the beginning and the end of the experience.

### Vestibulo-ocular reflex under normal visual feedback: vHIT and VR-HIT comparison

**Figure 4:**
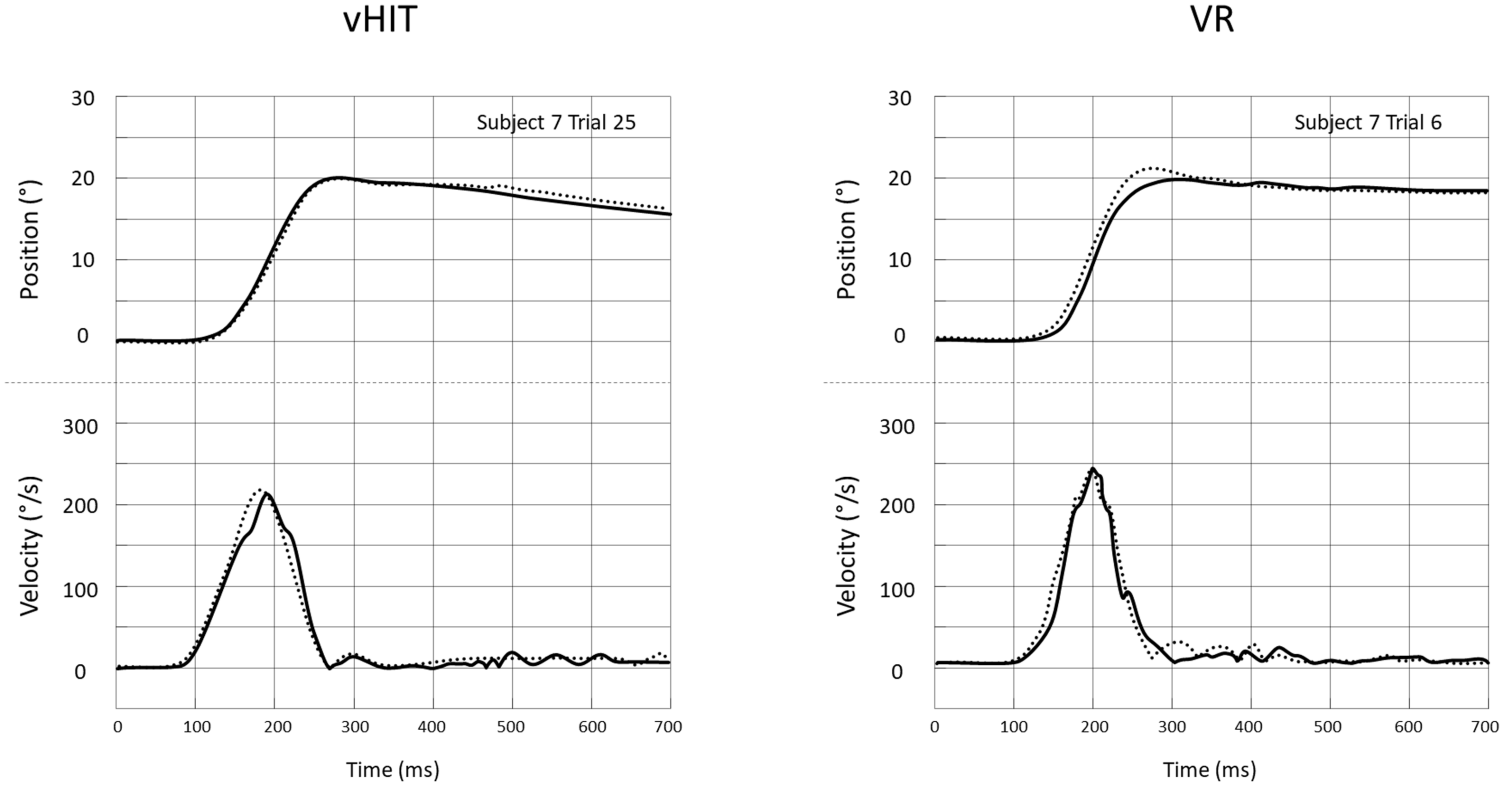
Head (^…….^) and eye (^___^) positions **(top)** and absolute velocities **(bottom)** for single head impulses recorded in the vHIT setting **(left)** and the VR-HIT setting **(right)**. Eye positions are inverted in the plot by convention. In both cases, recordings are from the same person and the same direction of HIT.

Recording of vHIT and VR-HIT were comparable in quality with few artefacts concerning eye or head movements in both methods. A comparison of a recording of head and eye movements during HIT for the same subject is shown in Figure 4.

**Figure 5:**
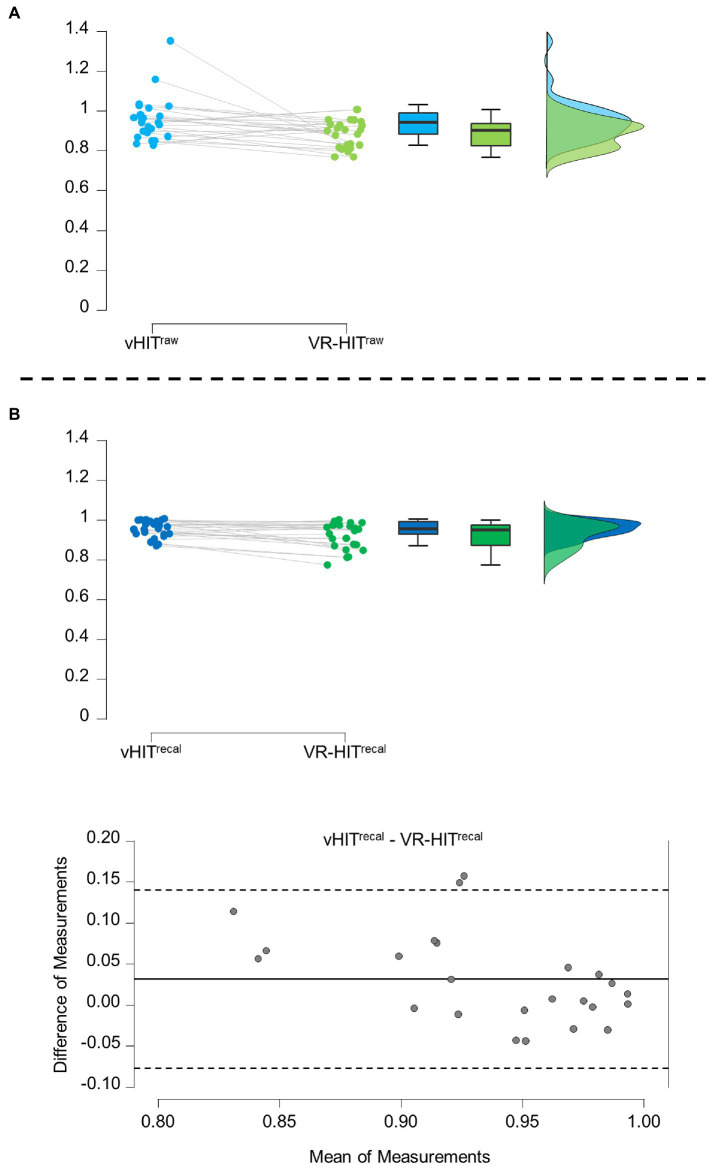
**(A)** VOR gain comparison between VR-HIT and vHIT for raw VOR (VORraw). Raincloud plot of VOR gain for vHIT (blue) and VR-HIT (green) during normal condition. The box plots show, from bottom to top, the lower extreme (excluding outliers), 1st quartile, mean, 3rd quartile, and upper extreme (excluding outliers). **(B)** VOR gain comparison between VR-HIT and vHIT for recalibrated VOR (VORrecal). Raincloud plot as described above and Bland–Altman plot are shown. In the Bland–Altman plot the mean difference is indicated by the solid line and the upper and lower 95% limits of agreement are indicated by the dashed line.

In the group of 12 subjects, the raw VOR gain (VOR^raw^) for vHIT and VR-HIT was, respectively, 0.96 (0.07) and 0.89 (0.11). For vHIT, 6 subjects had a VOR gain over 1, including one subject with a gain of 1.35 who did not produce any refixation saccade, thus indicating an eye-tracking calibration error (Figure 5). Thus, we calculated a recalibrated VOR gain (see Methods): VOR^recal^ was significantly higher in vHIT (0.95 +/− 0.04) than in VR-HIT (0.92 +/− SD 0.06) (Figure 5; Table 1). The mean gain of overt saccades was also significantly higher in VR-HIT compared to vHIT, in accordance with the lower VOR gain (Table 1). Finally, there was a significant higher head rotation speed, duration and amplitude using VR-HIT as compared to vHIT.

**Table 1:** Comparison of eye/head movement parameters between vHIT and VR-HIT under normal visual feedback.

	vHIT	VR-HIT	*p*
Recalibrated VOR gain	0.95 (0,04)	0.92 (0,07)	0.01
Covert saccades frequency (%)	1 (3)	2 (7)	0.23
Overt saccades frequency (%)	63 (38)	56 (37)	0.11
Overt saccades gain	0.05 (0.03)	0.10 (0.05)	<0.001
Head rotation duration (ms)	173 (13.2)	210 (22.6)	<0.001
Head rotation amplitude (°)	19.1 (2.3)	23.1 (2.3)	<0.001
Head rotation speed (°/s)	215 (13.6)	240 (30.4)	0.02

The group means and SD (parentheses) of each parameter are shown, followed by the p-value of the statistical comparisons [paired *t*-tests (*n*=12)].

Only one participant (age 72) had a VOR^recal^ gain lower than 0.8 for the VR-HIT setting (i.e., 0.77 on the right side, as compared to 0.89 in vHIT).

### VR in altered visual conditions

**Figure 6:**
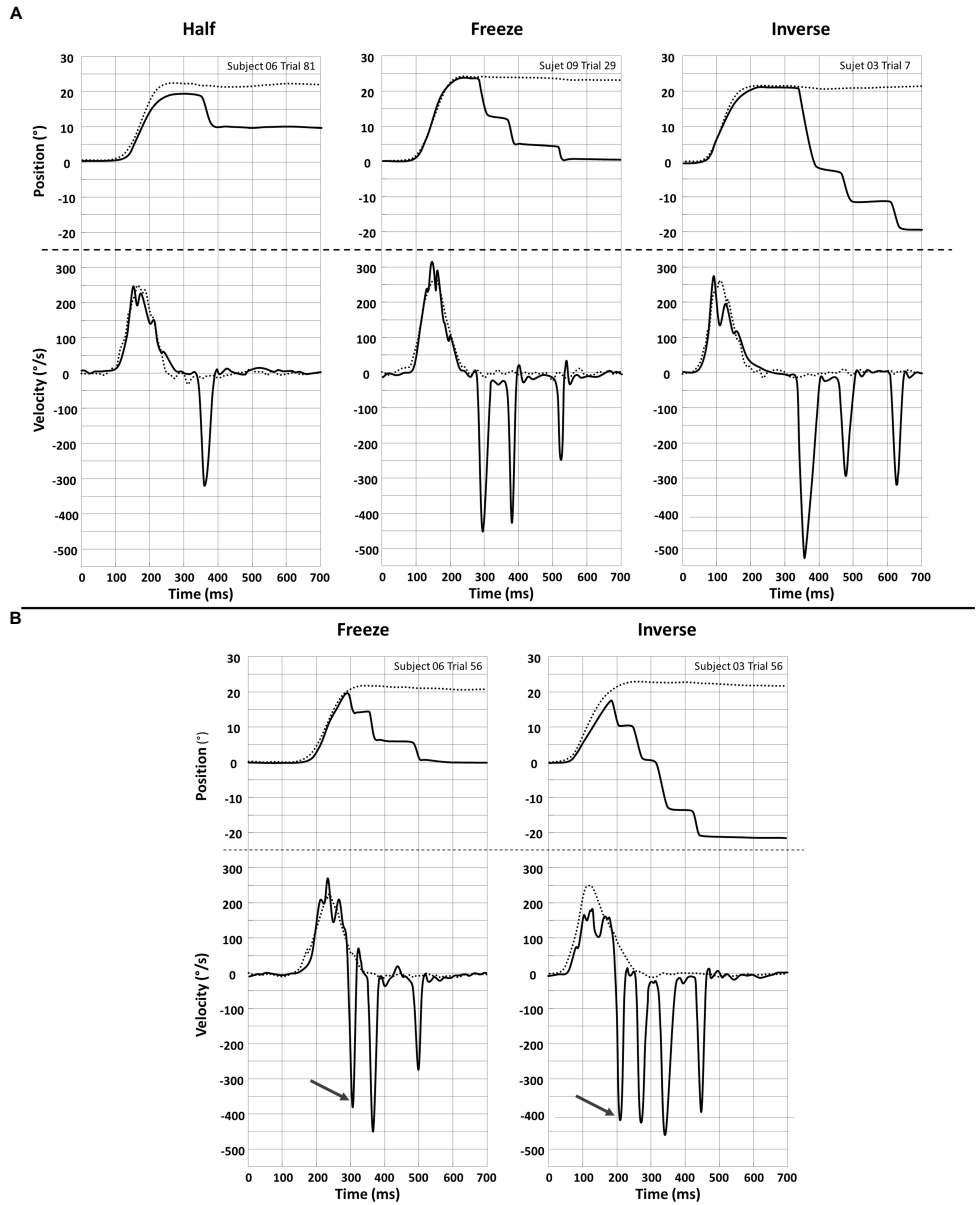
Recording of Head (^…….^) and eye (^___^) position (upper plots) and velocity (lower plots) for a single head impulse in the Half, Freeze and Inverse VR setting. Eye positions are inverted in the plot by convention. **(A)** Responses with overt saccades only. **(B)** Responses with a covert saccade (arrow) preceding overt saccades.

Examples of recording of eye and head movements during head impulses in the different conditions are shown in Figure 6. Responses including overt and covert saccades are shown in panels A and B, respectively. The shortest latency of all saccades recorded in the 12 subjects was 144 ms for Half and Freeze and 148 ms for Inverse.

In all examples (panels A & B), the superposition of head and eye positions clearly illustrate that, regardless of the type of visual information available to the participants, a high-gain VOR is triggered during the head impulses. Then, around 200 ms after the beginning of head movement (thus very close to its termination), an abrupt change in eye position is achieved by one -or more- saccade. These saccades are elicited in order to correct for the eye position error relative to the fixation point. This error depends on the mismatch condition as the intended final eye displacement equals: half of the head displacement and in the compensatory direction (−50%) for Half; 0% of the head displacement (central fixation) for Freeze, 100% of the head displacement but in the anti-compensatory angle for Inverse.

There was no significant effect of condition (Normal, Half, Freeze and Inverse) on Raw VOR gain in VR (ANOVA main effect of condition; *F* = 0.67; *p* = 0.570). There was no significant effect of altered visual conditions (Half, Freeze and Inverse) on other eye movements parameters except for the first saccade gain which was smaller in Half vs. Freeze vs. Inverse conditions (Table 2). This latter result could be linked to the progressive increase between these 3 conditions of gaze error achieved by the VOR at the end of the head movement.

**Table 2:** Effect of altered visual conditions on different oculomotor parameters.

	Half	Freeze	Inverse	*p*
Raw VOR gain	0.87 (0.08)	0.86 (0.06)	0.87 (0.07)	0.72
1st saccade gain	0.37 (0.08)	0.51 (0.17)	0.71 (0.28)	< 0.001
1st saccade latency (ms)	241 (39.4)	241 (40)	256 (57)	0.41
Covert saccades frequency (%)	25 (29)	27 (29)	31(33)	0.78
Covert saccades latency (ms)	187 (21,1)	196 (15.6)	198 (19.1)	0.25

The group means and SD (parentheses) of each parameter are shown, followed by the p-value of the main effect of condition assessed by ANOVA.

## Discussion

The main goal of this study was to describe the feasibility of eye and head movement measurement in a VR environment during high velocity horizontal head rotation in a normal condition. To our knowledge this is the first study trying to implement vHIT testing in a VR environment. Even though the mean residual vestibulo-ocular reflex in VR (0.92) was lower than during vHIT (0.95) our protocol seems to allow reliable head and eye velocity measurements in a VR setup during rapid head movements.

The secondary objective of the experiment was to describe the feasibility of creating different altered visual feedback and of recording eye and head movement in such context. Our results suggest that our setting allows for precise objective measurements in all 3 altered conditions.

### Eye tracking

Progress has been made regarding eye tracking in VR setups. In gaming, this eye tracking feature can be used to only render what is in the field of view of the player rather than the entire environment, allowing to limit computational burden. Eye tracking in VR can also be used in different research setups to evaluate attention and learning (17, 18). Nevertheless, native eye tracking systems still fall short regarding sampling rate as even the most recent commercially available devices are running at a 120 Hz frequency. This frequency is insufficient when analyzing VOR and saccades and standard vHIT setups indeed usually operate at 250 Hz. The use of a secondary implemented eye tracking system with a sampling rate of 250 Hz allowed us to overcome this shortcoming. Hopefully this additional Eye tracking device will not be needed in coming years if future generations of VR setups improve the recording frequency of native eye trackers.

### Head movement tracking

While native head motion tracking of VR setups can be used for standing balance (19) and slow movements (15), it could not be used for our protocol due to the high velocity of head movements. The artifact we observed could be due to build-in processing in the VR system which is implemented to reduce motion sickness and improve sense of presence. Since the raw data measured by the native gyroscope and accelerometer were encapsulated inside the HMD proprietary system and thus inaccessible, we decided to use another head tracking system.

We used a VICON setup which has shown sub-millimetric spatial resolution (13, 14). The reflective markers in our study were fixed on the HMD. The VR HMD markedly differs in weight from the vHIT goggles (respectively 470 g and 60 g) inducing a risk of slippage of the HMD relative to the head. When using vHIT goggles slippage is also a potential problem related to the weight of the setup and strap tightness (1, 20). To control for this during VR-HIT, we recorded head motion using both reflective markers fixed on the HMD and others fixed on a cap directly on the subject’s head. No lagging, overshooting, or bouncing were identified and no difference of recorded head trajectories were observed between the two setups. Slippage was thus considered to be negligible. Furthermore, we did not encounter any artefact which could be attributed to slippage during the rest of the study.

Another consequence of the weight of the VR setup still needs to be considered. Indeed, as the main differences between trials in vHIT and VR concerned the speed, amplitude and duration of the head movements, it is reasonable to assume that the weight of the HMD slightly affected the kinematics of head movements produced by the examiner.

### Potential spatial and temporal measurement errors due to VR

The latency between a real motion and its representation in the virtual scene is called motion-to-photon latency (M2P). A short M2P latency is needed in these experiments and especially in the visual mismatch conditions. The M2P latency is explained by a systematic process which can be subdivided in three major steps. First the head motion recorded by the integrated tracking system is collected by the Game Engine. Second, the different condition rendering effects are computed. Only this second step is accessible for optimization, the computational time depending on the complexity of the virtual scene. In our experiment, the complexity of the scene and the effects of the different visual feedback mismatch conditions were negligible. Therefore, the computational time was minimized and remained always smaller than the refresh time. In the third step, the rendered scene is transferred to the display through the pipeline renderer which stocks data in buffer to stabilize the frame-rate of the HMD display (21, 22).

From published data, the total M2P latency during a range of different head movements varied from 2 to 50 ms (23–25). This dynamic change in latency is suspected to be multifactorial, including a motion prediction mechanism that makes the virtual visual scene catch up with the real one in case of prolonged head rotations (21, 26). For the HMD we use the M2P latency has been measured to decrease from 30.8 ms at the onset of the movement to 3.6 ms over the course of 54 ms (26). These timeline of these mechanisms are however difficult to quantify as they are dependent on proprietary software of commercially available HMD. Thus the exact M2P latency of our setups is not known, however none of the subjects complained of a delay between head rotation and movement of the visual scene.

### Precision and reliability of VOR measurements

In our protocol, VOR gain in healthy subjects seems under-evaluated by 0.03 when comparing VR-HIT and vHIT. Even though this difference is statistically significant, it is relatively small (3.1%). In addition, in the VR setting, out of the 24 tested sides, all but one (VOR gain = 0.77) provided a VOR gain above the 0.80 threshold which is usually considered as a normal VOR gain for the lateral canals (26). These results have to be taken into account when using our setup.

As previously shown by many studies, VOR gain can adapt in case of mismatch between head motion and motion of the visual field. This adaptation can occur unilaterally and can be visible after only 15 min of training (6, 27) . In our virtual reality setup, we did not find any significant difference of VOR gain between the 4 conditions of visual feedback. This is probably due to the short period during which subjects were exposed to each mismatch condition and to the complete randomization of the conditions.

During our study, approximately 25% of trials were rejected manually due to artifacts mostly with regards to eye position. This was mostly observed when subjects blinked during head impulse but in some cases loss of signal was observed. This rejection rate is is similar to that of classical vHIT setups where this rejection process is automatic. We suggest increasing by 30% the number of intended head impulses when planning a study with the VR setup.

### Future applications of this setup

The use of VR setups in research and clinical applications has greatly increased in the last decade. This is also true with regards to studies of the vestibular system. In scientific setups, VR has been used for balance or spatial navigation explorations in patients with bilateral vestibular loss (10, 11). It is also frequently used for rehabilitation and physical therapy in patients with vestibular impairment (5, 6).

VR setups allow to create mismatch between head movement and resulting visual feedback in far more ecological conditions as compared to prisms or magnifying spectacles. This method could help to better explore VOR adaptation mechanisms both in healthy subjects and patients, thanks to the possibility of simultaneously manipulating head-motion visual feedback provided by ecologically-relevant virtual scenes and of quantifying the corresponding changes in eye movements response.

Many questions remain open with regards to compensation of VOR hypofunction and especially the development of *short latency catch up saccades* (or covert saccades). In particular, the neural mechanisms which actually trigger these saccades are still debated (28–30). The present VR set-up allowing to simultaneously record head and eye motion while creating different types of mismatch between head motion and visual feedback could give great insight into the role of visual information for triggering these *short latency catch up saccades*.

Finally, there is also a potential use of HIT recording during therapeutic VR setups such as modulation of VOR gain in physiotherapy requiring mismatch conditions. In the future this approach could eliminate the need for a second method of HIT recording permitting greater flexibility when monitoring progress of this physical therapy provided that native eye and head movement recording in VR will become more reliable.

## Data availability statement

The raw data supporting the conclusions of this article will be made available by the authors, without undue reservation.

## Ethics statement

The studies involving human participants were reviewed and approved by the National French ethical committee on human experimentation (ID-RCB: 2020-A00184-35). The patients/participants provided their written informed consent to participate in this study. Written informed consent was obtained from the individual(s) for the publication of any identifiable images or data included in this article.

## Author contributions

CD, GV, RS, AF, DP, CF, and RH designed the setup and the study. CD and RH performed experiments. CF and RH analyzed the data. CD, GV, and RH drafted the article. DP and CF critically reviewed and significantly contributed to the article. All authors provided critical feedback, helped shape the research, analyses, and article, and approved the final manuscript.

## Funding

This study was partially financed by a grant from the Association Française d’OtoNeurologie (AFON) Grand 2020.

## Conflict of interest

The authors declare that the research was conducted in the absence of any commercial or financial relationships that could be construed as a potential conflict of interest.

## Publisher’s note

All claims expressed in this article are solely those of the authors and do not necessarily represent those of their affiliated organizations, or those of the publisher, the editors and the reviewers. Any product that may be evaluated in this article, or claim that may be made by its manufacturer, is not guaranteed or endorsed by the publisher.
